# Care manager role for older multimorbid heart failure patients’ needs in relation to psychological distress and quality of life: a cross-sectional study

**DOI:** 10.3389/fcvm.2024.1432588

**Published:** 2024-09-30

**Authors:** Sara Gostoli, Regina Subach, Francesco Guolo, Francesco Bernardini, Alessandra Cammarata, Graziano Gigante, Birgit Herbeck Belnap, Diego Della Riva, Stefano Urbinati, Chiara Rafanelli

**Affiliations:** ^1^ Department of Psychology “Renzo Canestrari”, University of Bologna, Bologna, Italy; ^2^ Division of Cardiology, Bellaria Hospital, AUSL Bologna, Bologna, Italy; ^3^ Department of Psychosomatic Medicine and Psychotherapy, University of Göttingen Medical Centre, Göttingen, Germany; ^4^ Center for Behavioral Health, Media, and Technology, Division of General Internal Medicine, University of Pittsburgh School of Medicine, Pittsburgh, PA, United States; ^5^ Department of Psychology, University of Southern Denmark, Odense, Denmark

**Keywords:** care manager, patient’s needs, blended collaborative care, psychological distress, psychosomatic distress, somatization, quality of life

## Abstract

**Background:**

There are few studies investigating patients’ needs in healthcare focusing on disease severity and psychological characteristics of elderly heart failure (HF) patients with multimorbidity, specifically addressed by a care manager (CM).

**Aims:**

To explore the role of a CM dealing with elderly multimorbid HF patients’ needs/preferences according to NYHA class, ejection fraction, psychological/psychosomatic distress and quality of life (QoL), utilizing a Blended Collaborative Care (BCC) approach (ESCAPE; Grant agreement No 945377).

**Methods:**

Cue cards, self-reported questionnaires, and a semi-structured interview were used to collect data.

**Results:**

Twenty-five Italian patients (mean age ± *SD* = 77.5 ± 6.68) were enrolled between June 2021 and March 2022. The most relevant patients’ needs to be addressed by a CM were: education (e.g., on medical comorbidities), individual treatment tailoring (e.g., higher number of appointments with cardiologists) and symptom monitoring.

**Conclusion:**

The study highlights the importance of targeting HF patients’ needs according to psychological characteristics, whose healthcare requires person-centered care with CM assistance. In view of ESCAPE BCC intervention, a CM should consider specific patients’ needs of elderly multimorbid HF patients with psychological, psychosomatic distress, particularly somatization, and lower QoL to achieve a more personalized health care pathway.

**Study registration:**

The «Evaluation of a patient-centred biopsychosocial blended collaborative care pathway for the treatment of multi-morbid elderly patients» (ESCAPE) study has been registered at the University of Göttingen Medical Centre (UMG Reg. No 02853) and the German Clinical Trials Register (DRKS00025120).

## 1 Introduction

Elderly heart failure (HF) patients with multimorbidity usually experience both physical and psychological difficulties. Approximately 60% of elderly HF patients have at least three comorbidities, such as hypertension, heart rhythm disorders, chronic renal failure, diabetes mellitus and anemia (1). Furthermore, depressive symptoms are common among patients with HF, with prevalence rates ranging from 41.9% for any severity to 28.1% for moderate to severe (2). Therefore, elderly HF patients require specific health care based on their needs and preferences not only according to health-related goals. Specific needs may differ case by case and can be related to disease trajectory, symptom severity, and complexity of patients’ condition and psychological distress, such as depression and anxiety (3, 4). According to the literature, advanced New York Heart Association (NYHA) classes were found to be associated with needs of physical, psychological, social and existential support (5, 6), as well as needs related to acute symptoms management, information about the disease, alternative treatments, better communication with healthcare personnel and assistance with daily life activities (5–7). Moreover, preserved ejection fraction (EF) seems to be associated with specific treatment-related problems and needs, such as diagnostic difficulty, unclear illness perceptions and disease management complexity (8). Furthermore, existing literature underlines that psychosomatic distress, in particular allostatic overload (9), is associated with cardiovascular diseases, by affecting patients’ clinical course and outcomes (9, 10). Moreover, elderly HF patients with advanced NYHA classes report higher disheartenment within demoralization and lower quality of life (QoL) (11, 12).

The Collaborative Care (CC) approach which is based on the widely-used and evidence-based Chronic Care Model (CCM) (13) has been shown effective as a health care support for chronically ill patients such as those affected by HF, who often also present with somatic and mental comorbidities (14, 15). CC aims to guide patients through fragmented medical and nursing services, effectively enhance their self-management and use informal support services. On the basis of previous literature, the ESCAPE project defines a care manager (CM) as a trained health provider (typically a nurse) in different local healthcare settings, who plays a specific role in educating and discussing patient's care needs through a proactive, shared-decision approach to improve patients’ self-management. Specifically, a CM should implement individual care plan by integrating both patient's preferences and healthcare needs in prioritizing multiple treatments and behavior modification, monitoring symptoms, coordinating care across formal carers, connecting patients with external resources, motivating and supporting patients in behavioral change (16).

Pertaining to the Italian National Health Service, the role of a CM has not been widely implemented. Although a variety of models based on CCM have been studied across Italy (17–19), only few reported CM involvement in chronic care management of HF patients. For example, the “Leonardo Project” introduced a trained nurse as CM in primary healthcare settings in order to test the feasibility of disease and care management programs among patients with HF, as well as with other chronic illnesses. In this project, CM performed initial and follow-up assessments at patients’ home, educated patients on specific conditions, implemented individual care plan with consideration of patients health-related goals, assisted with service coordination and provided one-to-one coaching session to address individual patients’ concerns and aims (17). The study suggested that a cooperative and collaborative team of physicians, specialists, CMs and patients was beneficial for patients’ self-management skills, changes in health-related behaviors and increased patients’ disease-related knowledge (17).

Although a previous study within the ESCAPE project (20) acknowledged the potential role of the CM in collecting and addressing needs and preferences of elderly HF multimorbid patients in view of Blended Collaborative Care (BCC), to the best of our knowledge, there are no studies investigating the potential role of CMs in addressing those patients’ needs, according to HF severity and psychological burden.

## 2 Materials and methods

The aim of the present study was to explore the role of a CM in addressing patients’ needs and preferences according to disease severity (e.g., NYHA class and ejection fraction <40%), psychological/psychosomatic distress and QoL.

### 2.1 Design

This study represents an exploratory descriptive cross-sectional hypothesis-generating research. It refers to the Patient Public Involvement phase within the framework of a large, international and multi-center trial entitled “Evaluation of a patient-centered biopsychosocial blended collaborative care pathway for the treatment of multi-morbid elderly patients” (ESCAPE project; Horizon 2020; Grant Agreement No. 945377). The study has been registered at the University of Göttingen Medical Centre (UMG Reg. No. 02853) and the German Clinical Trials Register (DRKS00025120). The detailed description of ESCAPE project is available elsewhere (16). The present study followed the recommendations of the Declaration of Helsinki and was approved by all the local Ethics Committees. All participants were fully informed about the study, the voluntary nature of their participation, confidentiality and anonymity, and they all gave their written consent to participate.

### 2.2 Sample

Between June 2021 and March 2022, a convenience sample, including 25 elderly outpatients diagnosed with HF, was enrolled at the Division of Cardiology of Bellaria Hospital in Bologna (Italy). Inclusion criteria were: (a) clinically confirmed chronic HF, (b) at least two other somatic chronic comorbidities, (c) age ≥ 65 years, (d) written informed consent. Exclusion criteria were: (a) life expectancy less than one year due to other medical conditions than HF, (b) communication difficulties (speech, hearing, no means of contact such as telephone), (c) bipolar disorder, active suicidality, schizophrenia, and dementia. The local Ethics Committee approved the study.

### 2.3 Assessment

The assessment procedure in the current study was based on ESCAPE project protocol (16), supplemented by the inclusion of the Semi-Structured Interview based on the revised version of Diagnostic Criteria for Psychosomatic Research (DCPR-R) (16) to assess the presence of psychosomatic syndromes, such as allostatic overload and demoralization, which represented a deviation from the ESCAPE protocol.

#### 2.3.1 Anamnestic data

Socio-demographic data and clinical characteristics were collected by patient self-report with a specifically designed questionnaire, which included information on age, gender, residence, educational, marital and occupational status, as well as physical measurements (e.g., weight and height), number of comorbidities and duration of HF.

#### 2.3.2 Disease severity

NYHA classes were investigated through medical records consultation. The classification system categorizes HF into four classes, ranging from no symptoms and no physical activity limitations (Class I) to severe limitations with symptoms even at rest (Class IV).

#### 2.3.3 Systolic function

Ejection fraction has been defined as “low” (EF ≤ 40%), mid-range (41–49) and “preserved” (EF ≥ 50%) (21), and it has been collected through medical records.

#### 2.3.4 Psychological distress

Generalized Anxiety Disorder 7-item scale (GAD-7) (22), a self-rated measure to evaluate the severity of anxiety symptoms, was used. GAD-7 score can range from 0 to 21, since each of the 7 items can be scored from 0 (not at all) to 3 (almost every day). The total scores are categorized as follows: minimal/no anxiety (0–4), mild anxiety (5–9), moderate anxiety (10–14), or severe anxiety (15–21) (23). For the purposes of this study, a score of 5 was set as a cut-off to divide the sample into sub-groups according to the presence of “mild-severe anxiety” (≥5) vs. “no anxiety” (<5).

Patient Health Questionnaire, 9 items (PHQ-9) (24), a self-report measure to assess the severity of depression symptoms, was used. PHQ-9 score can range from 0 to 27, since each of the 9 items can be scored from 0 (not at all) to 3 (nearly every day) (24). The total scores are categorized as follows: minimal/no depression (0–4), mild depression (5–9), moderate depression (10–14), or severe depression (15–27) (25). For the purposes of this study, a score of 5 was set as a cut-off to divide the sample into sub-groups according to the presence of “mild-severe depression” (≥ 5) vs. “no depression” (<5).

#### 2.3.5 Psychosomatic distress

Patient Health Questionnaire, 15 items (PHQ-15) (26), a self-report measure to assess the severity of somatic symptoms, was used. PHQ-15 score can range from 0 to 30, since each of the 15 items can be scored from 0 (not bothered at all) to 2 (bothered at all). The total scores are categorized as follows: minimal/no somatization (0–4), mild somatization (5–9), moderate somatization (10–14), or severe somatization (15–30) (25). For the purposes of this study, a score of 5 was set as a cut-off to divide the sample into sub-groups according to the presence of “mild-severe somatization” (≥5) vs. “no somatization” (<5).

The Semi-Structured Interview based on the revised version of Diagnostic Criteria for Psychosomatic Research (DCPR-R) (27); to investigate allostatic overload and demoralization was used. The instrument reported good levels of reliability and validity (28), and the joint application of the DCPR and DSM-5 has been shown to improve the identification of psychological problems in patients with a variety of medical disorders (9, 29, 30).

#### 2.3.6 Quality of life

The European Quality of Life 5 Dimensions, 3 Level Version (EQ-5D-3L) (31), represents a self-report measure to assess quality of life according to five questions addressing mobility, self-care, usual activity, pain/discomfort and anxiety/depression. It consists of 5 items, each with response options on a Likert scale ranging from 1 (no problem) to 3 (extreme problems). The raw score is transformed in an index (from 0 to 1) in order to be compared with normative data, which were calculated among general population in five European countries, including Italy (32). An additional item, the European Quality Visual Analogue Scale (EQ-VAS), has been included in order to evaluate perceived health status. The respondent has to mark, in a vertical scale ranging from 0 (“the worst health you can imagine”) to 100 (“the best health you can imagine”), his/her current health status (31). For the purposes of this study, patients were allocated to “low” QoL/perceived health or “good” QoL/perceived health group, respectively, according to normative data stratified by age and sex (32).

#### 2.3.7 Patients’ needs

We used cue cards to identify patients’ needs that can be addressed by a CM, as well as an interview, to collect quantitative data. The description of the cards is detailed elsewhere (20). The themes of the cards are based on BCC intervention described by Herbeck Belnap et al. (33). In order to ask questions comprehensibly, the cards were presented according to 6 main topics: *education* (e.g., areas patients would like to get more information about, such as healthy lifestyle, different topics around the diseases, common HF comorbidities; preferred means of education), *individual tailoring* (e.g., areas patients would like their healthcare to be more personalized, such as medical treatment, personal life, ability to actively engage in improving own health, treatment burden), *monitoring* (e.g., aspects of the disease CM should keep an eye on, such as physical symptoms, medical information related to other conditions and personal life, medications prescription, adherence to treatment recommendations), *support* (e.g., areas in which patients need support by a CM, such as adaptation of treatment plan to daily life, health behaviors, emotional burden, communication with GP and/or informal carer), *coordination* (e.g., aspects a CM should help coordinate, such as updates to GP, collaboration with informal carer in patient's health management, finding specialists or community resources, specialist referrals, non-medical problems) and *communication* (e.g., means of communication with CM, such as in-person meetings, telephone, video calls or modern technologies) (33).

### 2.4 Statistical analyses

Data were analyzed using SPSS 20.0 (SPSS Inc., Chicago, IL) and the significance level was set at 0.05, two-tailed.

In the total sample, descriptive analyses were performed. Socio-demographic, medical and psychological characteristics, as well as patients’ needs and preferences, were presented as frequencies and/or means (*SD*). Concerning needs and preferences, only the first priority card was considered.

Chi-square test, applied to contingency tables, was used to compare different patients’ needs/preferences according to NYHA classes (I, II, III) and EF (<40% vs. ≥40%) and to evaluate the null hypothesis of independence between the different variables. If the test results to be significant (*p* < 0.05), then we have dependence between the two considered variables, otherwise there is no sufficient empirical evidence to reject the null hypothesis of independence.

Chi-square test, applied to contingency tables, was also used to compare different patients’ needs/preferences according to psychological, psychosomatic distress and QoL.

## 3 Results

### 3.1 Description of the sample

The present sample (*N* = 25) is characterized by 72% of men, with a mean age of 77.5 (*SD* = 6.68), ranging from 65 to 89 years. Regarding participants’ education, the average school attendance was 7.92 (*SD* = 3.89) years. Of all patients, 78.3% confirmed living with someone, whereas 21.7% lived alone. Most of the participants (76%) reported at least 3 comorbidities, 76% had NYHA II class and 40% had low (<40%) ejection fraction. All patients in the sample were prescribed with ≥5 medications, with 45.5% of them taking ≥10 medications. In terms of HF medications, 90.1% of the patients were prescribed diuretics, 54.6% beta-blockers, and 22.7% angiotensin receptor-neprilysin inhibitors (ARNIs). As for other common medications, the prevalence of cholesterol-lowering agents (77.3%), anticoagulants (72.7%), hyperuricemia (68%) medications, diabetes medications (63.6%), and proton pump inhibitors (59%) was also notable. Among all patients, 58.3% reported depression and anxiety. Regarding psychosomatic distress, 12% reported allostatic overload and 28% demoralization. The majority of the participants (88%) reported somatization. Only 12% of the patients self-reported low QoL, whereas 28% reported low perceived health status (Table 1).

**Table 1:** Sociodemographic, clinical and psychological characteristics of the sample (*N = 25*)*.*

Socio-demographic, clinical and psychological variables	*N* (%)	Mean ± SD
Age		77.5 ± 6.68
Gender		
Male	18 (72)	
Female	7 (28)	
Marital status		
Married	20 (80)	
Widow/widower	4 (16)	
Divorced	1 (4)	
Living condition		
Living with someone	18 (78.3)	
Living alone	5 (21.7)	
Occupational status		
Retired	25 (100)	
Years of schooling		7.92 ± 3.89
Number of comorbidities		
2	6 (24)	
3	8 (32)	
4	4 (16)	
5	7 (28)	
Height (cm)		169 ± 10.8
Weight (kg)		79.2 ± 19.5
Years with cardiac disease		10 ± 9.16
NYHA Class		
Class I	2 (8)	
Class II	19 (76)	
Class III	4 (16)	
Class IV	0 (0)	
Ejection fraction		
Low	10 (40)	
Mid-range	7 (28)	
Preserved	8 (32)	
Medications^a^		8.77 ± 2.25
<5	0 (0)	
5–9	12 (54.5)	
≥10	10 (45.5	
HF medications		
Diuretics	20 (90.1%)	
Beta-Blockers	12 (54.6%)	
Angiotensin receptor neprilysin inhibitors	5 (22.7%)	
Angiotensin receptor blockers	4 (18.2%)	
Angiotensin-converting-enzyme inhibitors	4 (18.2%)	
Other		
Cholesterol lowering medications	17 (77.3%)	
Anticoagulants	16 (72.7%)	
Hyperuricemia medications	15 (68%)	
Diabetes medications	14 (63.6%)	
Proton pump inhibitors	13 (59%)	
Psychological distress^b^		
Depression (PHQ-9)		
No distress	10 (41.7)	
From mild to severe distress	14 (58.3)	
Anxiety (GAD-7)		
No distress	10 (41.7)	
From mild to severe distress	14 (58.3)	
Psychosomatic distress		
Somatization (PHQ-15)		
No distress	3 (12)	
From mild to severe distress	22 (88)	
DCPR-R		
Allostatic overload	3 (12)	
Demoralization	7 (28)	
Quality of life		
EQ-5D-3L		
Low self-reported quality of life	3 (12)	
Good self-reported quality of life	22 (88)	
EQ-VAS		
Low perceived health status	7 (28)	
Good perceived health status	18 (72)	

EQ-5D-3L, European quality of life 5 dimensions, 3 Level version; EQ-VAS, European quality visual analogue scale; DCPR-R, diagnostic criteria for psychosomatic research, revised version; GAD-7, general anxiety disorder, 7 items; HF, heart failure; NYHA, New York heart association; PHQ-9, patient health questionnaire, 9 items; PHQ-15, patient health questionnaire, 15 items.

^a^ These data were calculated on sample *N* = 22, due to missing records.

^b^ These data were calculated on a sample of *N* = 24 because questionnaires of one patient were incomplete.

### 3.2 Patients’ needs

The most frequent patients’ needs within each area are presented in Table 2.

**Table 2:** Healthcare needs and preferences of the total sample (*N* = 25).

Healthcare needs and preferences	*N* (%)
Education	
Area to get more information about	
Healthy lifestyle	6 (24)
Topics around the disease	15 (60)
Common medical conditions	4 (16)
Most important subject within healthy lifestyle	
Sleep	5 (20)
Diet	3 (12)
Exercise	2 (8)
Stress	2 (8)
Not interested in the topic	13 (52)
Most important subject within topics around the disease	
Heart failure	18 (72)
Having several medical conditions at the same time	3 (12)
Not interested in the topic	4 (16)
Emotional distress	0 (0)
Most important subject within common medical conditions	
Diabetes type II	4 (16)
Osteoarthritis	3 (12)
Hypertension	1 (4)
Chronic obstructive pulmonary disease	1 (4)
Chronic kidney disease	1 (4)
Not interested in the topic	15 (60)
Favourite means of communication	
Education through care manager	21 (84)
Brochure by medical organizations	1 (4)
Text on a website	3 (12)
Video clip	0 (0)
Audio file	0 (0)
Individual tailoring	
Area of treatment to be more personalized	
Ability to actively engage in improving health	8 (32)
Personal life	5 (20)
Medical treatment	5 (20)
Treatment burden	4 (16)
Not interested in the topic	3 (12)
Most important subject within medical treatment	
More centralized treatment	3 (12)
Changes in treatment	1 (4)
Consideration of alternative treatments	0 (0)
Not interested in the topic	21 (84)
Most important subject within personal life	
Quality of life	4 (16)
Leisure time activities	1 (4)
Social life	0 (0)
Ability to work	0 (0)
Not interested in the topic	20 (80)
Most important subject within ability to actively engage in improving health	
Self-management	4 (16)
Engage in therapies	2 (8)
Physical activity	1 (4)
Change diet	1 (4)
Not interested in the topic	17 (68)
Most important subject within treatment burden	
Number of appointments	5 (20)
Different therapy modules	0 (0)
Not interested in the topic	20 (80)
Monitoring	
Aspect of diseases to be checked by care manager	
Symptoms	17 (68)
Medical information	3 (12)
Medication prescriptions	0 (0)
Following treatment recommendations	1 (4)
Not interested in the topic	4 (16)
Support	
Area to be supported by care manager	
Communication with GP/informal carer	6 (24)
Translation	6 (24)
Reduction of emotional burden	5 (20)
Health behaviours	2 (8)
Not interested in the topic	6 (24)
Coordination	
Aspect to be coordinated by care manager	
Updates	14 (56)
Assistance in finding necessary treatment specialists	2 (8)
Collaboration with informal carer in the management of health	1 (4)
Assistance in finding community resources	1 (4)
Non-medical	1 (4)
Specialist referrals	0 (0)
Not interested in the topic	6 (24)
Communication	
How to communicate with care manager?	
Telephone	12 (48)
Occasional in person meeting (together with GP)	9 (36)
Video call	1 (4)
Not interested in the topic	3 (12)
Other communication paths (modern technologies)	
Patient dashboard	0 (0)
Mobile phone messenger services	1 (50)
Patient dashboard with a communication function (e.g., emails) to care manager	0 (0)
Online chat forums	0 (0)
Not interested in the topic	24 (96)

GP, general practitioner.

### 3.3 Specific patients’ needs in association with disease severity, ejection fraction, psychological, psychosomatic distress and quality of life

According to disease severity (NYHA class and EF), patients did not differ in their report of patients’ needs to be directly or indirectly addressed by a CM (Table 3). However, depending on systolic function impairment (i.e., EF), most of the patients preferred to get information from CM about HF (*χ*^2^ = 12.8; *p* = 0.012) (Table 3).

**Table 3:** Needs And preferences according to disease severity: NYHA classes and ejection fraction (EF).

Healthcare needs and preferences	NYHA Class			*χ* ^2^	*p*	Ejection Fraction (EF)			*χ* ^2^	*p*
	I (*N* = 2)	II (*N* = 19)	III (*N* = 4)			Low (*N* = 10)	Mid-range (*N* = 7)	Preserved (*N* = 8)		
	*N* (%)	*N* (%)	*N* (%)			*N* (%)	*N* (%)	*N* (%)		
Education										
Area to get more information about				2.29	0.683				6.25	0.181
* *Healthy lifestyle	1 (50)	5 (23.3)	0 (0)			1 (10)	4 (57.1)	1 (12.5)		
* *Topics around the disease	1 (50)	11 (57.9)	3 (75)			7 (70)	2 (28.6)	6 (75)		
* *Common medical conditions	0 (0)	3 (15.8)	1 (25)			2 (20)	1 (14.3)	1 (12.5)		
* *Not interested in the topic	0 (0)	0 (0)	0 (0)			0 (0)	0 (0)	0 (0)		
Most important subject within healthy lifestyle				10.8	0.213				10.78	0.214
* *Exercise	1 (50)	1 (5.3)	0 (0)			0 (0)	1 (14.3)	1 (12.5)		
* *Diet	0 (0)	3 (15.8)	0 (0)			3 (30)	0 (0)	0 (0)		
* *Stress	0 (0)	1 (5.3)	1 (25)			0 (0)	1 (14.3)	1 (12.5)		
* *Sleep	1 (50)	4 (21.1)	0 (0)			1 (10)	3 (42.9)	1 (12.5)		
* *Not interested in the topic	0 (0)	10 (52.6)	3 (75)			6 (60)	2 (28.6)	5 (62.5)		
Most important subject within topics around the disease				7.53	0.110				12.84	0.012
* *Heart failure	2 (100)	14 (73.7)	2 (50)			8 (80)	3 (42.9)	7 (87.5)		
* *Emotional distress	0 (0)	0 (0)	0 (0)			0 (0)	0 (0)	0 (0)		
* *Having several medical conditions at the same time	0 (0)	1 (5.3)	2 (50)			2 (20)	0 (0)	1 (12.5)		
* *Not interested in the topic	0 (0)	4 (21.1)	0 (0)			0 (0)	4 (57.1)	0 (0)		
Most important subject within common medical conditions				17.3	0.068				9.42	0.493
* *Hypertension	0 (0)	0 (0)	1 (25)			0 (0)	0 (0)	1 (12.5)		
* *Diabetes type II	1 (50)	3 (15.8)	0 (0)			3 (30)	1 (14.3)	0 (0)		
* *Osteoarthritis	1 (50)	2 (10.5)	0 (0)			1 (10)	1 (14.3)	1 (12.5)		
* *Chronic obstructive pulmonary disease	0 (0)	1 (5.3)	0 (0)			0 (0)	1 (14.3)	0 (0)		
* *Chronic kidney disease	0 (0)	0 (0)	1 (25)			0 (0)	0 (0)	1 (12.5)		
* *Not interested in the topic	0 (0)	13 (68.4)	2 (50)			6 (60)	4 (57.1)	5 (62.5)		
Favourite means of communication				1.25	0.869				3.33	0.504
* *Text on a website	0 (0)	2 (10.5)	1 (25)			2 (20)	0 (0)	1 (12.5)		
* *Brochure by medical organizations	0 (0)	1 (5.3)	0 (0)			1 (10)	0 (0)	0 (0)		
* *Video clip	0 (0)	0 (0)	0 (0)			0 (0)	0 (0)	0 (0)		
* *Audio file	0 (0)	0 (0)	0 (0)			0 (0)	0 (0)	0 (0)		
* *Education through care manager	2 (100)	16 (84.2)	3 (75)			7 (70)	7 (100)	7 (87.5)		
* *Not interested in the topic	0 (0)	0 (0)	0 (0)			0 (0)	0 (0)	0 (0)		
Individual tailoring										
Area of treatment to be more personalized				3.31	0.913				5.86	0.663
* *Medical treatment	0 (0)	4 (21.1)	1 (25)			1 (10)	1 (14.3)	3 (37.5)		
* *Personal life	1 (50)	3 (15.8)	1 (25)			2 (20)	2 (28.6)	1 (12.5)		
* *Ability to actively engage in improving health	1 (50)	6 (31.6)	1 (25)			3 (30)	3 (42.9)	2 (25)		
* *Treatment burden	0 (0)	3 (15.8)	1 (25)			2 (20)	0 (0)	2 (25)		
* *Not interested in the topic	0 (0)	3 (15.8)	0 (0)			2 (20)	1 (14.3)	0 (0)		
Most important subject within medical treatment				1.50	0.826				3.98	0.409
* *Changes in treatment	0 (0)	1 (5.3)	0 (0)			1 (10)	0 (0)	0 (0)		
* *Consideration of alternative treatments	0 (0)	0 (0)	0 (0)			0 (0)	0 (0)	0 (0)		
* *More centralized treatment	0 (0)	3 (15.8)	0 (0)			0 (0)	1 (14.3)	2 (25)		
* *Not interested in the topic	2 (100)	15 (78.9)	4 (100)			9 (90)	6 (85.7)	6 (75)		
Most important subject within personal life				2.83	0.587				4.16	0.385
* *Quality of life	1 (50)	3 (15.8)	10 (0)			2 (20)	2 (28.6)	0 (0)		
* *Social life	0 (0)	0 (0)	0 (0)			0 (0)	0 (0)	0 (0)		
* *Ability to work	0 (0)	0 (0)	0 (0)			0 (0)	0 (0)	0 (0)		
* *Leisure time activities	0 (0)	1 (5.3)	0 (0)			1 (10)	0 (0)	0 (0)		
* *Not interested in the topic	1 (50)	15 (78.9)	4 (100)			7 (70)	5 (71.4)	8 (100)		
Most important subject within ability to actively engage in improving health				4.625	0.797				7.46	0.488
* *Physical activity	0 (0)	1 (5.3)	0 (0)			0 (0)	1 (14.3)	0 (0)		
* *Change diet	0 (0)	1 (5.3)	0 (0)			0 (0)	1 (14.3)	0 (0)		
* *Engage in therapies	0 (0)	1 (5.3)	1 (25)			1 (10)	1 (14.3)	0 (0)		
* *Self-management	1 (50)	3 (15.8)	0 (0)			2 (20)	1 (14.3)	1 (12.5)		
* *Not interested in the topic	1 (50)	13 (68.4)	3 (75)			7 (70)	3 (42.9)	7 (87.5)		
Most important subject within treatment burden				2.96	0.228				2.50	0.287
* *Number of appointments	0 (0)	3 (15.8)	2 (50)			3 (30)	0 (0)	2 (25)		
* *Different therapy modules	0 (0)	0 (0)	0 (0)			0 (0)	0 (0)	0 (0)		
* *Not interested in the topic	2 (100)	16 (84.2)	2 (50)			7 (70)	7 (100)	6 (75)		
Monitoring										
Aspect of diseases to be checked by care manager				9.07	0.170				4.93	0.553
* *Symptoms	1 (50)	14 (73.7)	2 (50)			6 (60)	4 (57.1)	7 (87.5)		
* *Medical information	1 (50)	2 (10.5)	0 (0)			1 (10)	2 (28.6)	0 (0)		
* *Medication prescriptions	0 (0)	0 (0)	0 (0)			0 (0)	0 (0)	0 (0)		
* *Following treatment recommendations	0 (0)	0 (0)	1 (25)			1 (10)	0 (0)	0 (0)		
* *Not interested in the topic	0 (0)	3 (15.8)	1 (25)			2 (20)	1 (14.3)	1 (12.5)		
Support										
Area to be supported by care manager				5.18	0.739				3.03	0.933
* *Translation	0 (0)	5 (26.3)	1 (25)			2 (20)	2 (28.6)	2 (25)		
* *Health behaviours	0 (0)	2 (10.5)	0 (0)			1 (10)	0 (0)	1 (12.5)		
* *Reduction of emotional burden	1 (50)	4 (21.1)	0 (0)			2 (20)	2 (28.6)	1 (12.5)		
* *Communication with GP/informal carer	0 (0)	4 (21.1)	2 (50)			2 (20)	1 (14.3)	3 (37.5)		
* *Not interested in the topic	1 (50)	4 (21.1)	1 (25)			3 (30)	2 (28.6)	1 (12.5)		
Coordination										
Aspect to be coordinated by care manager				5.80	0.832				8.90	0.541
* *Regular updates to GP	2 (100)	11 (57.9)	1 (25)			5 (50)	3 (42.9)	6 (75)		
* *Collaboration with informal carer in the management of health	0 (0)	1 (5.3)	0 (0)			0 (0)	1 (14.3)	0 (0)		
* *Assistance in finding necessary treatment specialists	0 (0)	1 (5.3)	1 (25)			1 (10)	0 (0)	1 (12.5)		
* *Assistance in finding community resources	0 (0)	1 (5.3)	0 (0)			1 (10)	0 (0)	0 (0)		
* *Specialist referrals	0 (0)	1 (5.3)	0 (0)			0 (0)	0 (0)	0 (0)		
* *Non-medical issues	0 (0)	1 (6.3)	0 (0)			0 (0)	1 (14.3)	0 (0)		
* *Not interested in the topic	0 (0)	4 (21.1)	2 (50)			3 (30)	2 (28.6)	1 (12.5)		
Communication										
How to communicate with care manager?				3.47	0.748				2.66	0.850
* *Occasional in person meeting (together with GP)	0 (0)	8 (42.1)	1 (25)			4 (40)	2 (28.6)	3 (37.5)		
* *Telephone	2 (100)	8 (42.1)	2 (50)			5 (50)	4 (57.1)	3 (37.5)		
* *Video call	0 (0)	1 (5.3)	0 (0)			0 (0)	0 (0)	1 (12.5)		
* *Not interested in the topic	0	2 (10.5)	1 (25)			1 (10)	1 (14.3)	1 (12.5)		
Other communication paths (modern technologies)				0.329	0.848				2.21	0.331
* *Patient dashboard	0 (0)	0 (0)	0 (0)			0 (0)	0 (0)	0 (0)		
* *Patient dashboard with a communication function (e.g., emails) to care manager	0 (0)	0 (0)	0 (0)			0 (0)	0 (0)	0 (0)		
* *Mobile phone messenger services	0 (0)	1 (5.3)	0 (0)			0 (0)	0 (0)	1 (12.5)		
* *Online chat forums	0 (0)	0 (0)	0 (0)			0 (0)	0 (0)	0 (0)		
* *Not interested in the topic	2 (100)	18 (94.7)	4 (100)			10 (100)	7 (100)	7 (87.5)		

GP, general practitioner; NYHA, New York heart association.

Among the six topics considered, patients experiencing psychological distress, psychosomatic complaints, and low QoL expressed different patients’ needs related to education, individual treatment tailoring and monitoring. These needs were to be directly or indirectly addressed by a CM (Tables 4–7).

**Table 4:** Needs and preferences according to psychological distress: depression (PHQ-9) and anxiety (GAD-7).

Healthcare needs and preferences	Depression		*χ* ^2^	*p*	Anxiety		*χ* ^2^	*p*
	Yes^a^ (*N* = 14)	No^b^ (*N* = 10)			Yes^c^ (*N* = 14)	No^d^ (*N* = 10)		
	*N* (%)	*N* (%)			*N* (%)	*N* (%)		
Education								
Area to get more information about			1.23	0.539			1.23	0.539
* *Healthy lifestyle	2 (14.3)	3 (30)			2 (14.3)	3 (30)		
* *Topics around the disease	10 (71.4)	5 (50)			10 (71.2)	5 (50)		
* *Common medical conditions	2 (14.3)	2 (20)			2 (14.3)	2 (20)		
* *Not interested in the topic	0 (0)	0 (0)			0 (0)	0 (0)		
Most important subject within healthy lifestyle			0.369	0.985			1.79	0.774
* *Exercise	1 (7.1)	1 (10)			2 (14.3)	0 (0)		
* *Diet	2 (14.3)	1 (10)			2 (14.3)	1 (10)		
* *Stress	1 (7.1)	1 (10)			1 (7.1)	1 (10)		
* *Sleep	2 (14.3)	2 (20)			2 (14.3)	2 (20)		
* *Not interested in the topic	8 (57.1)	5 (50)			7 (50)	6 (60)		
Most important subject within topics around the disease			6.40	0.041*			6.40	0.041*
* *Heart failure	11 (78.6)	7 (70)			11 (78.6)	7 (70)		
* *Emotional distress	0 (0)	0 (0)			0 (0)	0 (0)		
* *Having several medical conditions at the same time	3 (21.4)	0 (0)			3 (21.4)	0 (0)		
* *Not interested in the topic	0 (0)	3 (30)			0 (0)	3 (30)		
Most important subject within common medical conditions			5.49	0.360			5.78	0.328
* *Hypertension	1 (7.1)	0 (0)			0 (0)	1 (10)		
* *Diabetes type II	2 (14.3)	2 (20)			2 (14.3)	2 (20)		
* *Osteoarthritis	3 (21.4)	0 (0)			3 (21.4)	0 (0)		
* *Chronic obstructive pulmonary disease	0 (0)	1 (10)			0 (0)	1 (10)		
* *Chronic kidney disease	1 (7.1)	0 (0)			1 (7.1)	0 (0)		
* *Not interested in the topic	7 (50)	7 (70)			8 (57.1)	6 (60)		
Favourite means of communication			0.891	0.640			1.51	0.470
* *Text on a website	2 (14.3)	1 (10)			1 (7.1)	2 (20)		
* *Brochure by medical organizations	1 (7.1)	0 (0)			1 (7.1)	0 (0)		
* *Video clip	0 (0)	0 (0)			0 (0)	0 (0)		
* *Audio file	0 (0)	0 (0)			0 (0)	0 (0)		
* *Education through care manager	11 (78.6)	9 (90)			12 (85.7)	8 (80)		
* *Not interested in the topic	0 (0)	0 (0)			0 (0)	0 (0)		
Individual tailoring								
Area of treatment to be more personalized			7.07	0.132			7.07	0.132
* *Medical treatment	3 (21.4)	2 (20)			3 (21.4)	2 (20)		
* *Personal life	3 (21.4)	2 (20)			3 (21.4)	2 (20)		
* *Ability to actively engage in improving health	4 (28.6)	3 (30)			4 (28.6)	3 (30)		
* *Treatment burden	4 (28.6)	0 (0)			4 (28.6)	0 (0)		
* *Not interested in the topic	0 (0)	3 (30)			0 (0)	3 (30)		
Most important subject within medical treatment			1.51	0.470			0.891	0.640
* *Changes in treatment	1 (7.1)	0 (0)			1 (7.1)	0 (0)		
* *Consideration of alternative treatments	0 (0)	0 (0)			0 (0)	0 (0)		
* *More centralized treatment	1 (7.1)	2 (20)			2 (14.3)	1 (10)		
* *Not interested in the topic	12 (85.7)	8 (80)			11 (78.6)	9 (90)		
Most important subject within personal life			0.830	0.660			1.43	0.490
* *Quality of life	2 (14.3)	2 (20)			3 (21.4)	1 (10)		
* *Social life	0 (0)	0 (0)			0 (0)	0 (0)		
* *Ability to work	0 (0)	0 (0)			0 (0)	0 (0)		
* *Leisure time activities	1 (7.1)	0 (0)			1 (7.1)	0 (0)		
* *Not interested in the topic	11 (78.6)	8 (80)			10 (71.4)	9 (90)		
Most important subject within ability to actively engage in improving health			4.32	0.365			3.83	0.429
* *Physical activity	0 (0)	1 (10)			0 (0)	1 (10)		
* *Change diet	0 (0)	1 (10)			1 (7.1)	0 (0)		
* *Engage in therapies	2 (14.3)	0 (0)			2 (14.3)	0 (0)		
* *Self-management	2 (14.3)	1 (10)			2 (14.3)	1 (10)		
* *Not interested in the topic	10 (71.4)	7 (70)			9 (64.3)	8 (80)		
Most important subject within treatment burden			4.51	0.034*			4.51	0.034*
* *Number of appointments	5 (35.7)	0 (0)			5 (35.7)	0 (0)		
* *Different therapy modules	0 (0)	0 (0)			0 (0)	0 (0)		
* *Not interested in the topic	9 (64.3)	10 (100)			9 (64.3)	10 (100)		
Monitoring								
Aspect of diseases to be checked by care manager			0.887	0.828			2.88	0.410
* *Symptoms	10 (71.4)	7 (70)			11 (78.6)	6 (60)		
* *Medical information	1 (7.1)	1 (10)			1 (7.1)	1 (10)		
* *Medication prescriptions	0 (0)	0 (0)			0 (0)	0 (0)		
* *Following treatment recommendations	1 (7.1)	0 (0)			1 (7.1)	0 (0)		
* *Not interested in the topic	2 (14.3)	2 (20)			1 (7.1)	3 (30)		
Support								
Area to be supported by care manager			4.80	0.308			8.91	0.063
* *Translation	3 (21.4)	3 (30)			3 (21.4)	3 (30)		
* *Health behaviours	1 (7.1)	1 (10)			2 (14.3)	0 (0)		
* *Reduction of emotional burden	4 (28.6)	0 (0)			4 (28.6)	0		
* *Communication with GP/informal carer	4 (28.6)	2 (20)			4 (28.6)	2 (20)		
* *Not interested in the topic	2 (14.3)	4 (40)			1 (7.1)	5 (50)		
Coordination								
Aspect to be coordinated by care manager			4.54	0.475			3.80	0.579
* *Regular updates to GP	7 (50)	6 (60)			8 (57.1)	5 (50)		
* *Collaboration with informal carer in the management of health	0 (0)	1 (10)			1 (7.1)	0 (0)		
* *Assistance in finding necessary treatment specialists	2 (14.3)	0 (0)			1 (7.1)	1 (10)		
* *Assistance in finding community resources	1 (7.1)	0 (0)			1 (7.1)	0 (0)		
* *Specialist referrals	0 (0)	0 (0)			0 (0)	0 (0)		
* *Non-medical issues	1 (7.1)	0 (0)			1 (7.1)	0 (0)		
* *Not interested in the topic	3 (21.4)	3 (30)			2 (14.3)	4 (40)		
Communication								
How to communicate with care manager?			1.54	0.672			2.57	0.463
* *Occasional in person meeting (together with GP)	5 (35.7)	3 (30)			5 (35.7)	3 (30)		
* *Telephone	7 (50)	5 (50)			8 (57.1)	4 (40)		
* *Video call	0 (0)	1 (10)			0 (0)	1 (10)		
* *Not interested in the topic	2 (14.3)	1 (10)			1 (7.1)	2 (20)		
Other communication paths (modern technologies)			1.46	0.227			1.46	0.227
* *Patient dashboard	0 (0)	0 (0)						
* *Patient dashboard with a communication function (e.g., emails) to care manager	0 (0)	0 (0)			0 (0)	0 (0)		
* *Mobile phone messenger services	0 (0)	1 (10)			0 (0)	1 (10)		
* *Online chat forums	0 (0)	0 (0)			0 (0)	0 (0)		
* *Not interested in the topic	14 (100)	9 (90)			14 (100)	9 (90)		

GAD-7, general anxiety disorder, 7 items; GP, general practitioner; PHQ-9, patient health questionnaire, 9 items.

* *p* value <0.05.

^a^ PHQ-9 score ≥ 5.

^b^ PHQ-9 score < 5.

^c^ GAD-7 score ≥ 5.

^d^ GAD-7 score < 5.

**Table 5:** Needs and preferences according to psychosomatic distress: allostatic overload and demoralization (DCPR-R).

Healthcare needs and preferences	Allostatic overload		*χ* ^2^	*p*	Demoralization		*χ* ^2^	*p*
	Yes (*N* = 3)	No (*N* = 22)			Yes (*N* = 7)	No (*N* = 18)		
	*N* (%)	*N* (%)			*N* (%)	*N* (%)		
Education								
Area to get more information about			1.48	0.476			0.612	0.736
* *Healthy lifestyle	0 (0)	6 (27.3)			1 (14.3)	5 (27.8)		
* *Topics around the disease	2 (66.7)	13 (59.1)			5 (71.4)	10 (55.6)		
* *Common medical conditions	1 (33.3)	3 (13.6)			1 (14.3)	3 (16.7)		
* *Not interested in the topic	0 (0)	0 (0)			0 (0)	0 (0)		
Most important subject within healthy lifestyle			5.21	0.266			1.51	0.825
* *Exercise	0 (15.4)	2 (9.1)			0 (0)	2 (11.1)		
* *Diet	1 (33.3)	2 (9.1)			1 (14.3)	2 (11.1)		
* *Stress	1 (33.3)	1 (4.5)			1 (14.3)	1 (5.6)		
* *Sleep	0 (53.8)	5 (22.7)			1 (14.3)	4 (22.2)		
* *Not interested in the topic	1 (33.3)	12 (54.5)			4 (57.1)	9 (50)		
Most important subject within topics around the disease			1.85	0.396			9.57	0.008**
* *Heart failure	2 (66.7)	16 (72.7)			4 (57.1)	14 (77.8)		
* *Emotional distress	0 (0)	0 (0)			0 (0)	0 (0)		
* *Having several medical conditions at the same time	1 (33.3)	2 (9.1)			3 (42.9)	0 (0)		
* *Not interested in the topic	0 (0)	4 (18.2)			0 (0)	4 (22.2)		
Most important subject within common medical conditions			9.06	0.107			4.75	0.448
* *Hypertension	0 (0)	1 (4.5)			0 (0)	1 (5.6)		
* *Diabetes type II	1 (33.3)	3 (13.6)			1 (14.3)	3 (16.7)		
* *Osteoarthritis	0 (0)	3 (13.6)			0 (0)	3 (16.7)		
* *Chronic obstructive pulmonary disease	0 (0)	1 (4.5)			0 (0)	1 (5.6)		
* *Chronic kidney disease	1 (33.3)	0 (0)			1 (14.3)	0 (0)		
* *Not interested in the topic	1 (33.3)	14 (63.6)			5 (71.4)	10 (55.6)		
Favourite means of communication			7.86	0.020*			2.80	0.247
* *Text on a website	0 (0)	3 (13.6)			1 (14.3)	2 (11.1)		
* *Brochure by medical organizations	1 (33.3)	0 (0)			1 (14.3)	0 (0)		
* *Video clip	0 (0)	0 (0)			0 (0)	0 (0)		
* *Audio file	0 (0)	0 (0)			0 (0)	0 (0)		
* *Education through care manager	2 (66.7)	19 (86.4)			5 (71.4)	16 (88.9)		
* *Not interested in the topic	0 (0)	0 (0)			0 (0)	0 (0)		
Individual tailoring								
Area of treatment to be more personalized			7.95	0.093			5.90	0.207
* *Medical treatment	1 (33.3)	4 (18.2)			1 (14.3)	4 (22.2)		
* *Personal life	0 (0)	5 (22.7)			1 (14.3)	4 (22.2)		
* *Ability to actively engage in improving health	0 (0)	8 (36.4)			2 (28.6)	6 (33.3)		
* *Treatment burden	2 (66.7)	2 (9.1)			3 (42.9)	1 (5.6)		
* *Not interested in the topic	0 (0)	3 (13.6)			0 (0)	3 (16.7)		
Most important subject within medical treatment			1.55	0.460			0.435	0.805
* *Changes in treatment	0 (0)	1 (4.5)			0 (0)	1 (5.6)		
* *Consideration of alternative treatments	0 (0)	0 (0)			0 (0)	0 (0)		
* *More centralized treatment	1 (33.3)	2 (9.1)			1 (14.3)	2 (11.1)		
* *Not interested in the topic	2 (66.7)	19 (86.4)			6 (85.6)	15 (83.3)		
Most important subject within personal life			0.852	0.653			0.446	0.800
* *Quality of life	0 (0)	4 (18.2)			1 (14.3)	3 (16.7)		
* *Social life	0 (0)	0 (0)			0 (0)	0 (0)		
* *Ability to work	0 (0)	0 (0)			0 (0)	0 (0)		
* *Leisure time activities	0 (0)	1 (4.5)			0 (0)	1 (5.6)		
* *Not interested in the topic	3 (100)	17 (77.3)			6 (85.7)	14 (77.8)		
Most important subject within ability to actively engage in improving health			1.60	0.808			1.29	0.863
* *Physical activity	0 (0)	1 (4.5)			0 (0)	1 (5.6)		
* *Change diet	0 (0)	1 (4.5)			0 (0)	1 (5.6)		
* *Engage in therapies	0 (0)	2 (9.1)			1 (14.3)	1 (5.6)		
* *Self-management	0 (0)	4 (18.2)			1 (14.3)	3 (16.7)		
* *Not interested in the topic	3 (100)	14 (63.6)			5 (71.4)	12 (66.7)		
Most important subject within treatment burden			4.64	0.031*			8.38	0.004**
* *Number of appointments	2 (66.7)	3 (13.6)			4 (57.1)	1 (5.6)		
* *Different therapy modules	0 (0)	0 (0)			0 (0)	0 (0)		
* *Not interested in the topic	1 (33.3)	19 (86.4)			3 (42.9)	17 (94.4)		
Monitoring								
Aspect of diseases to be checked by care manager			1.60	0.658			4.87	0.182
* *Symptoms	3 (100)	14 (63.6)			4 (57.1)	13 (72.2)		
* *Medical information	0 (0)	3 (13.6)			0 (0)	3 (16.7)		
* *Medication prescriptions	0 (0)	0 (0)			0 (0)	0 (0)		
* *Following treatment recommendations	0 (0)	1 (4.5)			1 (14.3)	0 (20)		
* *Not interested in the topic	0 (0)	4 (18.2)			2 (28.6)	2 (11.1)		
Support								
Area to be supported by care manager			4.80	0.309			3.34	0.503
* *Translation	0 (0)	6 (27.3)			0 (0)	6 (33.3)		
* *Health behaviours	1 (33.3)	1 (4.5)			1 (514.3)	1 (5.6)		
* *Reduction of emotional burden	1 (33.3)	4 (18.2)			2 (28.6)	3 (16.7)		
* *Communication with GP/informal carer	1 (33.3)	5 (22.7)			2 (28.6)	4 (22.2)		
* *Not interested in the topic	0 (0)	6 (27.3)			2 (28.6)	4 (22.2)		
Coordination								
Aspect to be coordinated by care manager			2.68	0.749			3.39	0.641
* *Regular updates to GP	3 (100)	11 (100)			4 (57.1)	10 (55.6)		
* *Collaboration with informal carer in the management of health	0 (0)	1 (4.5)			0 (0)	1 (5.6)		
* *Assistance in finding necessary treatment specialists	0 (0)	2 (9.1)			0 (0)	2 (11.1)		
* *Assistance in finding community resources	0 (0)	1 (4.5)			0 (0)	1 (5.6)		
* *Specialist referrals	0 (0)	0 (0)			0 (0)	0 (0)		
* *Non-medical issues	0 (0)	1 (4.5)			0 (0)	1 (5.6)		
* *Not interested in the topic	0 (0)	6 (27.3)			3 (42.9)	3 (16.7)		
Communication								
How to communicate with care manager?			0.800	0.850			2.82	0.421
* *Occasional in person meeting (together with GP)	1 (33.3)	8 (36.4)			2 (28.6)	7 (38.9)		
* *Telephone	2 (66.7)	10 (45.5)			3 (42.9)	9 (50)		
* *Video call	0 (0)	1 (4.5)			0 (0)	1 (5.6)		
* *Not interested in the topic	0 (0)	3 (13.6)			2 (28.6)	1 (5.6)		
Other communication paths (modern technologies)			0.142	0.706			0.405	0.524
* *Patient dashboard	0 (0)	0 (0)			0 (0)	0 (0)		
* *Patient dashboard with a communication function (e.g., emails) to care manager	0 (0)	0 (0)			0 (0)	0 (0)		
* *Mobile phone messenger services	0 (0)	1 (4.5)			0 (0)	1 (5.6)		
* *Online chat forums	0 (0)	0 (0)			0 (0)	0 (0)		
* *Not interested in the topic	3 (100)	21 (95.5)			7 (100)	17 (94.4)		

DCPR-R, diagnostic criteria for psychosomatic research, revised version; GP, general practitioner.

* *p* value <0.05.

** *p* value <0.01.

**Table 6:** Needs and preferences according to psychosomatic distress: somatization (PHQ-15).

Healthcare needs and preferences	Somatization		*χ* ^2^	*p*
	Yes^a^ (*N* = 22)	No^b^ (*N* = 3)		
	*N* (%)	*N* (%)		
Education				
Area to get more information about			0.694	0.707
* *Healthy lifestyle	5 (22.7)	1 (33.3)		
* *Topics around the disease	13 (59.1)	2 (66.7)		
* *Common medical conditions	4 (18.2)	0 (0)		
* *Not interested in the topic	0 (0)	0 (0)		
Most important subject within healthy lifestyle			2.37	0.668
* *Exercise	2 (9.1)	0 (0)		
* *Diet	2 (9.1)	1 (33.3)		
* *Stress	2 (9.1)	0 (0)		
* *Sleep	4 (18.2)	1 (33.3)		
* *Not interested in the topic	12 (54.5)	1 (33.3)		
Most important subject within topics around the disease			1.06	0.558
* *Heart failure	16 (72.7)	2 (66.6)		
* *Emotional distress	0 (0)	0 (0)		
* *Having several medical conditions at the same time	3 (13.6)	0 (0)		
* *Not interested in the topic	3 (13.6)	1 (33.3)		
Most important subject within common medical conditions			2.27	0.810
* *Hypertension	1 (4.5)	0 (0)		
* *Diabetes type II	4 (18.2)	0 (0)		
* *Osteoarthritis	3 (13.6)	0 (0)		
* *Chronic obstructive pulmonary disease	1 (4.5)	0 (0)		
* *Chronic kidney disease	1 (4.5)	0 (0)		
* *Not interested in the topic	12 (54.5)	3 (100)		
Favourite means of communication			1.55	0.460
* *Text on a website	2 (9.1)	1 (33.3)		
* *Brochure by medical organizations	1 (4.5)	0 (0)		
* *Video clip	0 (0)	0 (0)		
* *Audio file	0 (0)	0 (0)		
* *Education through care manager	19 (86.4)	2 (66.7)		
* *Not interested in the topic	0 (0)	0 (0)		
Individual tailoring				
Area of treatment to be more personalized			10.4	0.034*
* *Medical treatment	5 (22.7)	0 (0)		
* *Personal life	5 (22.7)	0 (0)		
* *Ability to actively engage in improving health	7 (31.8)	1 (33.3)		
* *Treatment burden	4 (18.2)	0 (0)		
* *Not interested in the topic	1 (4.5)	2 (66.7)		
Most important subject within medical treatment			0.649	0.723
* *Changes in treatment	1 (4.5)	0 (0)		
* *Consideration of alternative treatments	0 (0)	0 (0)		
* *More centralized treatment	3 (13.6)	0 (0)		
* *Not interested in the topic	18 (81.8)	3 (100)		
Most important subject within personal life			0.852	0.653
* *Quality of life	4 (18.2)	0 (0)		
* *Social life	0 (0)	0 (0)		
* *Ability to work	0 (0)	0 (0)		
* *Leisure time activities	1 (4.5)	0 (0)		
* *Not interested in the topic	17 (77.3)	3 (100)		
Most important subject within ability to actively engage in improving health			1.19	0.880
* *Physical activity	1 (4.5)	0 (0)		
* *Change diet	1 (4.5)	0 (0)		
* *Engage in therapies	2 (9.1)	0 (0)		
* *Self-management	3 (13.6)	1 (33.3)		
* *Not interested in the topic	5 (68.2)	12 (66.7)		
Most important subject within treatment burden			0.852	0.356
* *Number of appointments	5 (22.7)	0 (0)		
* *Different therapy modules	0 (0)	0 (0)		
* *Not interested in the topic	17 (77.3)	3 (100)		
Monitoring				
Aspect of diseases to be checked by care manager			1.19	0.756
* *Symptoms	15 (68.2)	2 (66.7)		
* *Medical information	3 (13.6)	0 (0)		
* *Medication prescriptions	0 (0)	0 (0)		
* *Following treatment recommendations	1 (4.5)	0 (0)		
* *Not interested in the topic	3 (13.6)	1 (33.3)		
Support				
Area to be supported by care manager			4.48	0.345
* *Translation	5 (22.7)	1 (33.3)		
* *Health behaviours	2 (9.1)	0 (0)		
* *Reduction of emotional burden	5 (22.7)	0 (0)		
* *Communication with GP/informal carer	6 (27.3)	0 (0)		
* *Not interested in the topic	4 (18.2)	2 (66.7)		
Coordination				
Aspect to be coordinated by care manager			3.58	0.611
* *Regular updates to GP	13 (100)	0 (0)		
* *Collaboration with informal carer in the management of health	1 (4.5)	1 (33.3)		
* *Assistance in finding necessary treatment specialists	2 (9.1)	0 (0)		
* *Assistance in finding community resources	1 (4.5)	0 (0)		
* *Specialist referrals	0 (0)	0 (0)		
* *Non-medical issues	1 (4.5)	0 (0)		
* *Not interested in the topic	4 (18.2)	2 (66.7)		
Communication				
How to communicate with care manager?			2.90	0.407
* *Occasional in person meeting (together with GP)	9 (40.9)	0 (0)		
* *Telephone	10 (45.5)	2 (66.7)		
* *Video call	1 (4.5)	0 (0)		
* *Not interested in the topic	2 (9.1)	1 (33.3)		
Other communication paths (modern technologies)			0.142	0.706
* *Patient dashboard	0 (0)	0 (0)		
* *Patient dashboard with a communication function (e.g., emails) to care manager	0 (0)	0 (0)		
* *Mobile phone messenger services	1 (4.5)	0 (0)		
* *Online chat forums	0 (0)	0 (0)		
* *Not interested in the topic	21 (95.5)	3 (100)		

GP, general practitioner; PHQ-15, patient health questionnaire, 15 items.

* *p* value <0.05.

^a^ PHQ-15 score ≥ 5.

^b^ PHQ-15 score < 5.

**Table 7:** Needs and preferences according to self-reported quality of life (EQ-5D-3L) and perceived health status (EQ-VAS).

Healthcare needs and preferences	Self-reported quality of life		*χ* ^2^	*p*	Perceived health status		χ^2^	*p*
	Low (*N* = 3)	Good (*N* = 22)			Low (*N* = 7)	Good (*N* = 18)		
	*N* (%)	*N* (%)			*N* (%)	*N* (%)		
Education								
Area to get more information about			1.17	0.558			6.07	0.048*
* *Healthy lifestyle	1 (33.3)	5 (22.7)			2 (28.6)	4 (22.2)		
* *Topics around the disease	1 (33.3)	14 (63.6)			2 (28.6)	13 (72.2)		
* *Common medical conditions	1 (33.3)	3 (13.6)			3 (42.8)	1 (5.6)		
* *Not interested in the topic	0 (0)	0 (0)			0 (0)	0 (0)		
Most important subject within healthy lifestyle			3.95	0.413			1.81	0.770
* *Exercise	0 (0)	2 (9.1)			0 (0)	2 (11.1)		
* *Diet	0 (0)	3 (13.6)			1 (14.3)	2 (11.1)		
* *Stress	1 (3.33)	1 (4.5)			1 (14.3)	1 (5.6)		
* *Sleep	1 (3.33)	4 (18.2)			2 (28.6)	3 (16.7)		
* *Not interested in the topic	1 (3.33)	12 (54.5)			3 (42.9)	10 (55.6)		
Most important subject within topics around the disease			9.74	0.008*			2.54	0.281
* *Heart failure	1 (33.3)	17 (77.3)			4 (57.1)	14 (77.8)		
* *Emotional distress	0 (0)	0 (0)			0 (0)	0 (0)		
* *Having several medical conditions at the same time	2 (66.7)	1 (4.5)			2 (28.6)	1 (5.6)		
* *Not interested in the topic	0 (0)	4 (18.2)			1 (14.3)	3 (16.7)		
Most important subject within common medical conditions			8.59	0.127			6.07	0.300
* *Hypertension	0 (0)	1 (4.5)			0 (0)	1 (5.6)		
* *Diabetes type II	0 (0)	4 (18.2)			1 (14.3)	3 (16.7)		
* *Osteoarthritis	0 (0)	3 (13.6)			1 (14.3)	2 (11.1)		
* *Chronic obstructive pulmonary disease	0 (0)	1 (4.5)			1 (14.3)	0 (0)		
* *Chronic kidney disease	1 (33.3)	0 (0)			1 (14.3)	0 (0)		
* *Not interested in the topic	2 (66.7)	13 (59.1)			3 (42.9)	12 (66.7)		
Favourite means of communication			0.649	0.723			0.435	0.805
* *Text on a website	0 (0)	3 (13.6)			1 (14.3)	2 (11.1)		
* *Brochure by medical organizations	0 (0)	1 (4.6)			0 (0)	1 (5.6)		
* *Video clip	0 (0)	0 (0)			0 (0)	0 (0)		
* *Audio file	0 (0)	0 (0)			0 (0)	0 (0)		
* *Education through care manager	3 (100)	18 (81.8)			6 (85.7)	15 (83.3)		
* *Not interested in the topic	0 (0)	0 (0)			0 (0)	0 (0)		
Individual tailoring								
Area of treatment to be more personalized			7.24	0.124			7.02	0.135
* *Medical treatment	0 (0)	5 (22.7)			2 (28.6)	3 (16.7)		
* *Personal life	0 (0)	5 (22.7)			1 (14.3)	4 (22.2)		
* *Ability to actively engage in improving health	1 (33.3)	7 (31.8)			1 (14.3)	7 (38.9)		
* *Treatment burden	2 (66.7)	2 (9.1)			3 (42.8)	1 (5.6)		
* *Not interested in the topic	0 (0)	3 (13.6)			0 (0)	3 (16.7)		
Most important subject within medical treatment			0.649	0.723			2.80	0.247
* *Changes in treatment	0 (0)	1 (4.5)			1 (14.3)	0 (0)		
* *Consideration of alternative treatments	0 (0)	0 (0)			0 (0)	0 (0)		
* *More centralized treatment	0 (0)	3 (13.6)			1 (14.3)	2 (11.1)		
* *Not interested in the topic	3 (100)	18 (81.8)			5 (71.4)	16 (88.9)		
Most important subject within personal life			0.852	0.653			2.68	0.262
* *Quality of life	0 (0)	4 (18.2)			1 (14.3)	3 (16.7)		
* *Social life	0 (0)	0 (0)			0 (0)	0 (0)		
* *Ability to work	0 (0)	0 (0)			0 (0)	0 (0)		
* *Leisure time activities	0 (0)	1 (4.5)			1 (14.3)	0 (0)		
* *Not interested in the topic	3 (100)	17 (77.3)			5 (71.4)	15 (83.3)		
Most important subject within ability to actively engage in improving health			3.55	0.470			5.74	0.219
* *Physical activity	0 (0)	1 (4.5)			1 (14.3)	0 (0)		
* *Change diet	0 (0)	1 (4.5)			0 (0)	1 (5.6)		
* *Engage in therapies	1 (33.3)	1 (4.5)			0 (0)	2 (11.1)		
* *Self-management	0 (0)	4 (18.2)			0 (0)	4 (22.2)		
* *Not interested in the topic	2 (66.7)	15 (68.2)			6 (85.7)	11 (61.1)		
Most important subject within treatment burden			13.6	<.001**			3.17	0.075
* *Number of appointments	3 (100)	2 (9.1)			3 (42.9)	2 (11.1)		
* *Different therapy modules	0 (0)	0 (0)			0 (0)	0 (0)		
* *Not interested in the topic	0 (0)	20 (90.9)			4 (57.1)	16 (88.9)		
Monitoring								
Aspect of diseases to be checked by care manager			8.29	0.040*			0.466	0.926
* *Symptoms	2 (66.7)	15 (68.2)			5 (71,4)	12 (66.7)		
* *Medical information	0 (0)	3 (13.6)			1 (14.3)	2 (11.1)		
* *Medication prescriptions	0 (0)	0 (0)			0 (0)	0 (0)		
* *Following treatment recommendations	1 (33.3)	0 (0)			0 (0)	1 (5.6)		
* *Not interested in the topic	0 (0)	4 (18.2)			1 (14.3)	3 (16.7)		
Support								
Area to be supported by care manager			4.48	0.345			7.64	0.106
* *Translation	1 (33.3)	5 (22.7)			2 (28.6)	4 (22.2)		
* *Health behaviours	0 (0)	2 (9.1)			0 (0)	2 (11.1)		
* *Reduction of emotional burden	0 (0)	5 (22.7)			0 (0)	5 (27.5)		
* *Communication with GP/informal carer	2 (66.7)	4 (18.2)			4 (57.1)	2 (11.1)		
* *Not interested in the topic	0 (0)	6 (27.3)			1 (14.3)	5 (27.5)		
Coordination								
Aspect to be coordinated by care manager			0.875	0.972			4.21	0.519
* *Regular updates to GP	2 (66.7)	12 (54.5)			3 (42.9)	11 (61.1)		
* *Collaboration with informal carer in the management of health	0 (0)	1 (4.5)			0 (0)	1 (5.6)		
* *Assistance in finding necessary treatment specialists	0 (0)	2 (9.1)			1 (14.3)	1 (5.6)		
* *Assistance in finding community resources	0 (0)	1 (4.5)			1 (14.3)	0 (0)		
* *Specialist referrals	0 (0)	0 (0)			0 (0)	0 (0)		
* *Non-medical issues	0 (0)	1 (4.5)			0 (0)	1 (5.6)		
* *Not interested in the topic	1 (33.3)	5 (22.7)			2 (28.6)	4 (22.2)		
Communication								
How to communicate with care manager?			3.69	0.297			0.750	0.861
* *Occasional in person meeting (together with GP)	0 (0)	9 (40.9)			2 (28.6)	7 (38.9)		
* *Telephone	3 (100)	9 (40.9)			4 (57.1)	8 (44.4)		
* *Video call	0 (0)	1 (4.5)			0 (0)	1 (5.6)		
* *Not interested in the topic	0 (0)	3 (13.6)			1 (14.3)	2 (11.1)		
Other communication paths (modern technologies)			0.142	0.706			0.405	0.524
* *Patient dashboard	0 (0)	0 (0)			0 (0)	0 (0)		
* *Patient dashboard with a communication function (e.g., emails) to care manager	0 (0)	0 (0)			0 (0)	0 (0)		
* *Mobile phone messenger services	0 (0)	1 (4.5)			0 (0)	1 (5.6)		
* *Online chat forums	0 (0)	0 (0)			0 (0)	0 (0)		
* *Not interested in the topic	3 (100)	21 (95.5)			7 (100)	17 (94.4)		

EQ-5D-3L, European quality of life 5 dimensions, 3 Level version; EQ-VAS, European quality visual analogue scale; GP, General Practitioner.

* *p* value <0.05.

** *p* value <0.01.

*** *p* value <0.001.

Specifically, as to the topic of education, most of the patients with anxiety (78.6%) and depression (78.6%) preferred to get information on their comorbidities in addition to HF (*χ*^2^ = 6.40; *p* = 0.041) (Table 4). With regards to the topic of individual tailoring, even though all the patients without psychological distress (100%) and more than a half of patients with depression (64.3%) and anxiety (64.3%) were not interested in personalizing their treatment in terms of addressing treatment burden (*χ*^2^ = 4.51; *p* = 0.034), all the participants who were interested in the mentioned issue reported depression and anxiety; in particular, these patients asked to get a higher number of appointments with their cardiologists (Table 4).

As to psychosomatic distress, compared with patients without DCPR demoralization who mainly (77.8%) expressed the need for more information exclusively on HF (topic of education), demoralized participants were interested in getting information not only on HF (57.1%), but also regarding their multimorbidity (42.9%) (*χ*^2^ = 9.57, *p* = 0.008). Moreover, as to the topic of individual tailoring, even though 42.9% of demoralized patients were not interested in personalizing their treatment in terms of addressing treatment burden, 80% of those participants who would like more frequent appointments with cardiologists presented with demoralization (*χ*^2^ = 8.38; *p* = 0.004) (Table 5). On the same vein, two-thirds of patients with DCPR allostatic overload (66.7%) expressed the need of a higher number of visits (*χ*^2^ = 4.64; *p* = 0.031) (Table 5). Furthermore, this sub-group of patients would like to receive health-related information through brochure by medical organization (topic of education), in addition to education through CM him/herself, differently from patients without the same syndrome who preferred text on a website in addition to in-person education by CM (*χ*^2^ = 7.86; *p* = 0.020) (Table 5). Finally, compared with participants without somatic complaints, patients with PHQ-15 somatization were more interested in tailoring their treatment (*χ*^2^ = 10.4, *p* = 0.034), in particular in learning how to improve their own ability to actively enhance their health (31.8%) (Table 6).

Compared with most of the patients (77.3%) with good self-reported QoL (EQ-5D-3L) who preferred education on HF, two-thirds of those with low QoL required information about having several medical conditions at the same time (*χ*^2^ = 9.74, *p* = 0.008) (Table 7). Moreover, patients with low self-reported QoL expressed the need of increasing the frequency of appointments with the cardiologists (*χ*^2^ = 13.6; *p* < 0.001) (topic of individual tailoring), and having physical symptoms (e.g., shortness of breath, weight gain, blood pressure, glucose, LDL and other lab results) and treatment recommendation adherence monitored by CM (*χ*^2^ = 8.29, *p* = 0.040) (Table 7). Finally, compared with patients with good perceived health status (EQ-VAS) who mainly required more information about issues related to their medical condition (72.2%), those with low perceived health status reported heterogeneous educational needs concerning also comorbidities (such as hypertension, type II diabetes, osteoarthritis, chronic obstructive pulmonary disease, chronic kidney disease) and lifestyle (*χ*^2^ = 6.07; *p* = 0.048) (Table 7).

## 4 Discussion

To our knowledge few studies investigated HF patient's needs and preferences within a BCC approach, including recognition of CM role in collecting patients’ needs and preferences (20). However, no studies explored and described CM's role in addressing those healthcare needs according to disease severity, psychological, psychosomatic distress and QoL, in elderly HF patients with multimorbidity. Our study is the first one that tries to fill this gap in the literature.

In contrast to existing literature, in our study no significant differences in patients’ preferences and needs in association with NYHA classes were reported. However, the majority of the patients in the current study belonged to NYHA II class, whereas in the literature differences in needs were reported mainly between I-II and III-IV classes (5, 6). The results regarding EF showed that HF patients, based on their EF category, preferred receiving education and information about their condition from a CM. These findings are consistent with previous research, indicating that HF patients have treatment-related preferences linked to their EF, its associated symptoms, and management needs (8). Indeed, patients with low EF, which is associated with symptoms such as chest pain, heart palpitations, coughing (sometimes with blood), fatigue, and dizziness, might have different health management needs and preferences compared to those with mid-range or preserved EF. Additionally, the observed differences may be attributed to the higher prevalence of low EF among younger men compared to preserved EF, which is more commonly reported among older women (34).

Patients with psychological and psychosomatic distress, as well as low QoL, reported patients’ needs to be addressed by CM related to education, individual treatment tailoring, and monitoring. Most of the patients, especially those with psychological distress, demoralization and low self-reported QoL preferred to tailor their treatment with CM assistance by getting a higher number, in terms of frequency, of appointments with the cardiologists and receiving information about HF or their medical comorbidities, such as hypertension, diabetes type II, osteoarthritis, chronic obstructive pulmonary disease, chronic kidney disease, in addition to HF. According to the literature (35, 36), patients with a chronic disease re-evaluate the importance of communication with their GP, especially in adaptation of their treatment plans into their daily life and living conditions, due to perceived loss of independence. Presence of comorbidities is associated with psychological distress and decreased QoL (37). Since in our study the majority of the patients presented at least three comorbidities, this may explain why the patients requested from CM additional information about them.

Patients with allostatic overload preferred to receive health-related information also from brochures by medical organizations, in addition to education by CM. The results are in line with existing literature that suggests that allostatic overload is associated with cognitive loss, in particular with advanced age (38, 39), which could result in difficulties in memorizing and processing information related to complex treatment plans. Also, patients with low self-reported QoL preferred CM's support regarding symptom monitoring, such as shortness of breath, weight gain, blood pressure, glucose, LDL and other lab results, in addition to assistance with following treatment recommendations. The results of the present study are also in line with existing literature, as majority of the elderly patients reported at least 3 comorbidities in addition to HF that is associated with poor treatment coordination and reduced health-related quality of life (40, 41).

Despite that psychological and psychosomatic distress, as well as low QoL are common among elderly HF multimorbid patients, according to our knowledge there is a lack of information about CM's role in addressing these patients’ needs and preferences. The results of the current study highlight the importance of targeting patients with their needs whose healthcare requires person-centered care (42) with CM assistance. CM inclusion in complex treatment plans that address somatic and mental comorbidities are suggested to benefit quality of the provided health care (43) and increase patients’ satisfaction with treatment management. Furthermore, coordinated by a nurse, healthcare for elderly and multimorbid HF patients is suggested to be effective by RCTs in US (14). However, in the Italian healthcare system comparable support by a CM is not widely available. Instead, in Italy, usually nurses, as case managers, assist patients with acute conditions at home-care level, in order to prevent further progression of patients’ illness (19, 44). Only the “Leonardo Project” tested the use of CMs in a BCC approach, who assisted patients with multiple chronic conditions, supported them in following complex treatment plans, but only for a 18 month period (17).

Finally, in the present study patients with psychological or psychosomatic distress seem to belong to two separate categories, those who are not interested in taking actions to improve their healthcare vs. those patients who expressed specific needs to be addressed by a CM. This may support findings from another qualitative international study of elderly multimorbid HF patients’ healthcare needs (45) that identified three main patients’ profiles: those who need and want support; those who actively engage in self-care and only reach out to the health care system when needed; those who feel neglected by the health care system and do not believe in professional support. The lack of interest showed by some of the patients in the present study could thus be related to the latter two profiles.

### 4.1 Limitations

Limitations of this study include a relatively small sample size that cannot be considered representative of all elderly multimorbid patients with HF. Moreover, the utilization of a convenience sampling strategy resulted in a predominance of males and absence of patients with NYHA class IV heart failure, thereby limiting the generalizability and applicability of the study findings. We acknowledge that the significance of the chi-square test results also could have been influenced by the small sample size. However, this study is preliminary and exploratory in nature and it does not intend to establish definitive correlations; instead, it aimed at informing ESCAPE-recruited CMs, in order to help them in delivering BCC. Due to a cross-sectional design of the study, this research acquires data at a singular time point, therefore constraining the capacity to establish the potential evolution of patients’ needs and preferences over time. Additionally, the absence of a control group poses difficulties to ascertain whether the identified needs and preferences are specific to elderly HF patients or common among elderly individuals with other chronic conditions. Lastly, the monocentricity of study could have affected the study's external validity. Despite all these limitations the present study gives the opportunity to focus on the role a CM may play in addressing needs in relation to severity of disease, psychological, psychosomatic distress and QoL in elderly HF patients with multimorbidity.

### 4.2 Conclusion

In the Italian healthcare setting, the introduction of the CM role, supported by a Specialist Team, looks promising for enhancing the care of elderly HF patients. Future studies should consider patients’ needs and preferences considering the cited associations. The results of this study may also inform researchers interested in developing holistic treatments for elderly patients suffering from multiple physical and mental health conditions.

## Data Availability

The data that support the findings of this study will be available from the corresponding author, CR, upon reasonable request.
