# Supplementary material for: Care manager role for older multimorbid heart failure patients’ needs in relation to psychological distress and quality of life: a cross-sectional study
Source: Front Cardiovasc Med. 2024 Sep 30;11:1432588. doi: 10.3389/fcvm.2024.1432588 (PMC11475247; doi:10.3389/fcvm.2024.1432588)
Supplement: Supplementary file 1 [file Table1.pdf]

Supplementary Material

Care manager role for older multimorbid heart failure patients' needs in relation to psychological distress and quality of life: a cross- sectional study.

Sara Gostoli†, Regina Subach†, Francesco Guolo, Francesco Bernardini, Alessandra Cammarata, Graziano Gigante, Birgit Herbeck Belhap, Diego Della Riva, Stefano Urbinati, Chiara Rafanelli1*, On behalf of the ESCAPE Consortium

***Correspondence:** Corresponding Author: chiara.rafanelli@unibo.it

Table 1. ESCAPE Consortium list

No	Name	Short name	Country	Principal investigator	Authors of the current publication	Further members of the ESCAPE consortium
1	Syddansk Universitet	SDU<br>Odense	Denmark	• Prof. Susanne S. Pedersen<br>• Jens Søndergaard		• Trine Thilsing<br>• Susanne S. Pedersen<br>• Sanne Rasmussen<br>• Jens Søndergaard<br>• Sussi Friis Buhl
2	Region Sjaelland	ZEALCO - Slagelse<br>ZEALCO - Roskilde	Denmark	• Prof. Søren T. Skou<br>• Prof. Niels Eske Bruun		• Peter H. Gæde<br>• Mette Nyberg<br>• Mette Dideriksen<br>• Lars H. Tang<br>• Prof. Søren T. Skou<br>• Christian Axel Bang<br>• Gitte E. Ingwersen<br>• Prof. Niels Eske Bruun

3	Universitaetsmedizin Goettingen - Georg-August-Universitaet Goettingen - Stiftung Oeffentlichen Rechts	UMG	Germany	Prof. Christoph Herrmann-Lingen	• Birgit Herbeck Belnap	• Cornelia Regner<br>• Miriam Sass<br>• Christine von Arnim<br>• Mohammed Chebbok<br>• Michael Koziolek<br>• Kristina Lang<br>• Sandrin Plewe<br>• Anja Zinke<br>• Angela Knauf<br>• Ralf Tostmann<br>• Aaron Marshall<br>• Tina Krüger<br>• Christoph Herrmann-Lingen<br>• Christine Zelenak<br>• Jonas Nagel<br>• Kristina Bersch<br>• Tim Friede
4	Universitaetsklinikum Hamburg-Eppendorf	UKE	Germany	• Prof. Dr. med. Martin Scherer<br>• Prof. Bernd Löwe		• Hendrika Wiedemann<br>• Thomas Asendorf<br>• Florian Walker<br>• Dagmar Lühmann<br>• Sebastian Kohlmann<br>• Josefine Schulze<br>• Agata Menzel<br>• Prof. Dr. med. Martin Scherer<br>• Prof. Bernd Löwe

5	Klinikum der Universitaet zu Koeln	UHC	Germany	Prof. Christian Albus		• Christian Albus<br>• Anna Markser<br>• Lisa Derendorf<br>• Stephanie Stock<br>• Dusan Simic<br>• Dirk Müller
6	Universitaet Leipzig	ULEI	Germany	Dr. Rolf Wachter		• Liska Hoppe<br>• Irina Müller-Kozarez<br>• Rolf Wachter
7	Royal College of Surgeons in Ireland<br><br>Beaumont Hospital	RCSI	Ireland	Prof. Dr. Frank Doyle		RCSI:<br>• Frank Doyle<br>• Jan Sørensen<br><br>Beaumont Hospital:<br>• Brendan McAdam<br>• David Farrell<br>• Helen Claire Cooney
8	Lietuvos Sveikatos Mokslu Universitetas	LSMU	Lithuania	Dr. Margarita Beresnevaite		• Leonas Valius<br>• Romaldas Maciulaitis<br>• Egle Rumbinaite<br>• Margarita Beresnevaite
9	Semmelweis University	SEG	Hungary	• Dr. Klaudia Vivien Nagy<br>• Dr. Adrienne Stauder		• PhD Piroska Balog<br>• Zsuzsa Bernáth-Lukács<br>• Dr. Andrea Székely<br>• Adrienne Stauder<br>• Klaudia Vivien Nagy
10	Azienda Unità Sanitaria Locale (AUSL) di Bologna	BEL	Italy	Dr. Stefano Urbinati	• Stefano Urbinati<br>• Diego Della Riva	• Barbara Bordoni

					• Francesco Guolo	
11	Alma Mater Studiorum - Università di Bologna	UNIBO - University of Bologna	Italy	Prof. Chiara Rafanelli	• Chiara Rafanelli<br>• Sara Gostoli<br>• Regina Subach<br>• Graziano Gigante	
12	Fraunhofer Gesellschaft zur Foerderung der Angewandten Forschung E.V.	Fraunhofer	Germany	Dr. Carlos A Velasco		• Martin Breidenbach<br>• Florim Hamiti<br>• Aynur Guluzade<br>• Naguib Heiba<br>• Yehya Mohamad<br>• Carlos A Velasco
13	Eurocarers - Association Europeenne Travaillant avec et pour les Aidants Non-Professionnels	EAC	Belgium	Svetlana Atanasova		• Svetlana Atanasova<br>• Stecy Yghemonos<br>• Olivier Jacqmain
14	Bundesarbeitsgemeinschaft Selbsthilfe von Menschen mit Behinderung und Chronischer Erkrankung und ihren Angehorigen E.V.	BAG Selbsthilfe	Germany	Dr. Martin Danner		• Franziska Hetzer<br>• Martin Danner<br>• Christina Homma
15	Linkopings Universitet	LIU	Sweden	Prof. Tiny Jaarsma		• Tiny Jaarsma<br>• Frida Andréasson
16	ERINN Innovation	ERINN	Ireland	Rochelle Caruso		• Rochelle Caruso<br>• Jane Maher
17	Yale University New Haven	YALE	USA			• Matthew M. Burg

This table lists all consortium members who have contributed to the presented work.
